# Considerations for using race and ethnicity as quantitative variables in medical education research

**DOI:** 10.1007/s40037-020-00602-3

**Published:** 2020-08-12

**Authors:** Paula T. Ross, Tamera Hart-Johnson, Sally A. Santen, Nikki L. Bibler Zaidi

**Affiliations:** 1 University of Michigan—Michigan Medicine, Ann Arbor, MI USA; 2 Virginia Commonwealth School of Medicine, Richmond, VA USA

**Keywords:** Race, Ethnicity, Research, Quantitative methods

## Abstract

Throughout history, race and ethnicity have been used as key descriptors to categorize and label individuals. The use of these concepts as variables can impact resources, policy, and perceptions in medical education. Despite the pervasive use of race and ethnicity as quantitative variables, it is unclear whether researchers use them in their proper context. In this Eye Opener, we present the following seven considerations with corresponding recommendations, for using race and ethnicity as variables in medical education research: 1) Ensure race and ethnicity variables are used to address questions directly related to these concepts. 2) Use race and ethnicity to represent social experiences, not biological facts, to explain the phenomenon under study. 3) Allow study participants to define their preferred racial and ethnic identity. 4) Collect complete and accurate race and ethnicity data that maximizes data richness and minimizes opportunities for researchers’ assumptions about participants’ identity. 5) Follow evidence-based practices to describe and collapse individual-level race and ethnicity data into broader categories. 6) Align statistical analyses with the study’s conceptualization and operationalization of race and ethnicity. 7) Provide thorough interpretation of results beyond simple reporting of statistical significance. By following these recommendations, medical education researchers can avoid major pitfalls associated with the use of race and ethnicity and make informed decisions around some of the most challenging race and ethnicity topics in medical education.

## Background

Race and ethnicity are two of the most commonly used variables in research. A Google Scholar search of “medical education” and “race” or “ethnicity” yields more than 64,000 results; therefore, it is important to consider how they are used in medical education research to create new knowledge. The use of these variables in medical education research has wide-ranging implications in that any associated findings can shape perceptions of the groups under study, influence the allocation of resources, and impact implementation of new policy [1, 2]. These variables also have the potential to provide information that can address some of the most challenging topics in the field, including efforts to 1) promote diversity and inclusion of faculty, staff, and medical trainees; 2) identify incidents of racism, bias, and discrimination in the learning environment; and 3) address inequities in salary or access to education [3–7].

Race and ethnicity are frequently used interchangeably, despite having distinct meanings. The concept of race alone holds variable meanings. It is most often used as a biomedical indicator based on shared physical traits (e.g. skin color, facial features, hair texture, etc.) [8]. It is also used to refer to cultural patterns or to indicate socioeconomic conditions [8]. When used in this way, race becomes a connotation of different life experiences, social practices, and behaviors among a group of people who share a distinct sociocultural context [2]. Ethnicity is understood as an indication of shared cultural traditions, beliefs, history, celebrations, and language [8].

For over three centuries, race and ethnicity have served as one of the primary ways to categorize and label human differences in our society [8, 9]; however, these labels are ever-changing and nebulous. Much of the current categorization of race and ethnicity in the United States has been driven by the Office of Management and Budget and the U.S. Census Bureau, although these categories have changed with each census. The Association of American Medical Colleges (AAMC) continues to blur the distinctions between race and ethnicity, as it asks medical school applicants to self-identify by selecting race or ethnicity as a distinct category and/or in combination [10].

Despite the lack of biological evidence to support the concepts of race and ethnicity, the intentional use of these variables is necessary in order to generate accurate interpretation of research findings, prevent additional bias, and mitigate perpetuating stereotypes—particularly among vulnerable and racialized groups [11–13]. Other scientific disciplines (e.g., epidemiology, occupational therapy, public health, health services, biomedicine, and clinical practice) have addressed the use of race and ethnicity as variables and determined best practices in their respective fields [11, 14–20]. While biomedical research has attempted to better define race and ethnicity through the National Standards for Culturally and Linguistically Appropriate Services in Health and Health Care, medical education has yet to follow suit [21]. We suggest that when using these concepts as study variables, medical education researchers begin with a clear understanding of their intended meaning, purpose, and contribution to the study. Below we present seven considerations and recommendations for using race and ethnicity as quantitative variables in medical education research.

### Considerations for using race and ethnicity as quantitative research variables

#### Justify the inclusion of race and ethnicity as a research variable

Race and ethnicity variables should be used to explore phenomena directly related to these concepts, not as a proxy for other, unmeasured concepts (e.g., socioeconomic status, cultural factors, family structure, etc.) [2, 11, 17, 22]. As illustrated in Tab. 1, race and ethnicity can mediate or moderate other variables or, in some cases, describe differences in experiences among racial and ethnic groups.

**Table 1:** Justifications for including race (R) and ethnicity (E) variables in research

Role of the R & E variable	Purpose of R & E variable	Sample medical education research question using R & E variables
Grouping	To examine similarities or differences between R or E groups and/or subgroups based on a dependent (outcome) variable	Is there a significant difference in medical students’ access to professional mentors by R or E group?
Mediating	To examine whether R or E explains the relationship between an independent (predictor) and dependent (outcome) variable	Is the association between socioeconomic status and students’ perceptions of the medical school learning environment reduced when R or E are considered?
Moderating	To examine whether the strength of the relationship between an independent (predictor) and dependent variable (outcome) varies by R or E groups	Does the relationship between social support and well-being vary by R or E group?

##### Recommendation:

Clarify the purpose and justification for including race or ethnicity variables *a priori* [23]. Limit the use of these variables to topics in which they can directly contribute to the production of new knowledge about the studied phenomenon [24]. Even when research funding requires the inclusion of race and ethnicity data, researchers should continue to use these variables in their appropriate context. The following questions are helpful for considering appropriate use of race or ethnicity as a variable: How are race and ethnicity relevant to the study? How will the inclusion of race and ethnicity as a variable improve our understanding of the phenomenon under study?

#### Use race and ethnicity variables to explore social issues and experiences

Race and ethnicity variables must be situated within the appropriate context [2]. As noted above, race and ethnicity are concepts that capture experiences, rather than biological facts [25]. These concepts are not static or absolute but remain fluid in how individuals are labeled—both by society and by self. Therefore, using race and ethnicity to explain biomedical phenomena obscures the complex role in shaping an individual’s social identity and assigns false attribution to genetic factors [22]. When the research question explores an area of systemic racial or ethnic bias, race and ethnicity variables are appropriate.

Within the context of medical education, race and ethnicity variables are often used to discuss differences in academic performance such as grade point average (GPA). In this situation, relationships between GPA and racial and ethnic groups may be spurious if they are not causally related, or may be mediated by sociocultural factors such as bias in the content of standardized tests [26, 27]. GPA is an indicator of academic achievement rather than a product of social experience, yet misguided suggestions of causation may result in inaccurate attribution between race and ethnicity and academic performance. Thus, researchers may ignore potential contributing social factors and thereby misattribute results or behaviors with individuals’ intelligence or aptitude.

##### Recommendation:

Align race and ethnicity variables with the research question to explore experiences related to social phenomena, such as access to educational and professional resources or experiences within the learning environment (see examples in Tab. 1). Avoid the use of race and ethnicity to explore physiological or biological abilities or differences (e.g., intelligence, surgical dexterity, etc.), [14] since any differences could likely represent spurious correlations or cultural bias.

#### Collect rich and accurate race and ethnicity data

Many racial and ethnic categories reflect approximations of broad and overlapping socially defined groups based on shifting criteria; therefore, it is important to ensure that the data used for research purposes represents the population(s) under study as accurately as possible [28]. Data collection methods should maximize data accuracy and minimize researchers’ assumptions about participants’ race or ethnicity. Because physical characteristics or other factors (e.g. names) cannot be used as a proxy for race or ethnicity, observer-determined categorization may be subject to bias or error. Researchers commonly convert open-ended data into distinct, quantitative categories to facilitate statistical analysis. This requires the researcher to make decisions regarding data interpretation, calibration, and coding [29].

##### Recommendation:

Allow participants to indicate the racial and ethnic categories that best align with their personal experience. This information should therefore be participant-collected rather than observer-collected. Use a combination of pre-defined response categories and open-ended questions to ensure consistent and comprehensive methods for collecting race and ethnicity data [30].

Well-supported data collection strategies help mitigate researcher bias and assumptions of within-group homogeneity [9]. Therefore, we recommend researchers collect race and ethnicity data as separate items of inquiry, since these are distinct concepts [9]. When these variables are collected separately, it is not necessary for researchers to interpret the data. Perhaps the most successful and well-known strategy for collecting these data is the one adopted by the U.S. Census in 2000 which asks about Hispanic origin as a separate question, independent of race [31].

Ask participants to report the racial and ethnic background of their parents in addition to their self-classification, country of origin among all races (e.g., African American, Arab American, Asian, Hispanic, White, etc.)[17], and language(s) spoken at home. This practice will also help classify multi-racial individuals who may only identify with a single group [32]. Researchers can also use intentional sampling strategies (e.g., over sampling with correction via weighting) to ensure the study sample represents the intended population.

Finally, when reporting race and ethnicity data in figures and tables, include descriptors such as “self-reported data” to clarify the source of the information [11, 16]. Advantages and disadvantages for commonly used formats for collecting race and ethnicity data are included in Tab. 2.

**Table 2:** Advantages and disadvantages of various data collection methods

Category type	Advantage(s)	Disadvantage(s)	Example
**Multiple-response (exclusive)** **categories:** Multiple options provided; respondent can only select ONE pre-established category [33]	Maintains original unit(s) of analysis Provides more complete and accurate data [34] Aligns data with most statistical analyses [33] Permits respondents to self-report identity and allows researchers to collect rich data [35]	Provides less data per category which increases the risk of error in interpreting outcomes [36] Forces respondents into discrete category that does not allow for fluid or broad self-identification [37]	Respondent must select *one* option from White, African American, American Indian, Alaska Native, and Native Hawaiian
**Multiple-response (inclusive)** **categories:** Multiple options provided; respondent can select MULTIPLE options from pre-established categories [33]		Introduces issues related to comparability of samples across multiple data sets [38] Forces researcher to decide how individuals fit into certain categories [37] Counts multiracial respondents as members of each individual racial or ethnic group they select which inflates the number of respondents in denominator [33]	Respondent may select *multiple* options from White-non-Hispanic, African American, American Indian, Alaska Native, and Native Hawaiian
**Combined categories**^a^: Multiple options combined to define new categories	Simplifies statistical analysis, interpretation and presentation of results [39] Increases cell size when discrete categories are too small [40]	Limits conclusions to broad assumptions and generalizations about respondents within groups [41, 42] Perpetuates obsolete majority/minority discourse when using certain binary frameworks (e.g., White/non-White) [34] Uses subjective labels that can perpetuate bias/stereotypes [43] Increases the risk of a false positive result [44] Underestimates the extent of variation between groups by not fully accounting for within group variability [26]	Respondent must select *either* URiM^b^ [45] or Non-URiM

^a^Combined categories also can represent collapsed and dichotomous categories

^b^URiM Underrepresented in medicine

#### Use evidence-based practices to manipulate individual-level race and ethnicity data into categories

Ideally, datasets used for research should include an adequate representative sample of all racial and ethnic groups under study; however, this level of representation is not always available. In such cases, researchers may collapse data into fewer categories. When a specific racial and ethnic category has too few respondents, researchers manipulate discrete race and ethnicity data into broader categories, which may limit data analysis and impact the meaning of the results.

##### Recommendation:

If data must be manipulated, use evidence-based data strategies to collapse or combine data to ensure categorizations properly represent the groups or subgroups included in the sample. The study’s sample size, or cell size, of racial and ethnic subgroups should dictate data reduction strategies. The categories into which race and ethnicity data are collapsed should fit within the parameters of the research question(s) [46]. For example, the AAMC created the underrepresented in medicine (URiM) identification to acknowledge “racial and ethnic populations that are underrepresented in the medical profession relative to their numbers in the general population” [45]. Rather than specifying static racial and ethnic groups with this categorization, URiM allows for the inclusion or removal of groups based on changing local or national demographics [45]. While this identifier may address some study questions related to this topic, combining racial and ethnic categories may prevent researchers from examining the impact on specific groups.

#### Align statistical analyses with how variables were conceptualized and operationalized

Some research questions can only be addressed by conducting specific statistical analyses, and some analyses can only be conducted with certain types of data. Data analyses are dependent on how race and ethnicity were conceptualized in the research question and how variable(s) were operationalized (e.g., multiple-response or combined categorizations) [47]. Race and ethnicity are descriptive, rather than objectively measurable, concepts; therefore, they only change with subjective manipulation of the variables’ definitions and/or criteria for inclusion and are rarely used as dependent (or outcome) variables.

Race and ethnicity variables are categorical (or discontinuous) variables used to illustrate variation in labels rather than variation in level (ordinal or interval variables). Categorical variables are generally presented descriptively as frequencies and/or percentages, and analysis using race and ethnicity data is generally limited to statistical tests applicable for categorical data (e.g., T‑tests, ANOVA). Although, if the research question requires analysis that only utilizes continuous or dichotomous variables, a set of “dummy variables” can be created to describe the sample participants—understanding each participant should only be represented by one of those variables. When statistically significant differences are found between or within racial and ethnic groups, further exploration is necessary to determine whether the difference is based on an overriding variable of interest, or caused by variation within a specific race or ethnicity subgroup (e.g. non-Hispanic subgroup) [17].

##### Recommendation:

Align statistical analyses of race and ethnicity data with the research question(s) to ensure sufficient examination of the intended phenomena [11]. When possible, conduct the appropriate comparisons to examine whether significant differences are within or between racial and ethnic groups.

#### Provide a thorough interpretation of the results

Researchers may stratify data by race or ethnicity but fail to interpret or discuss their findings based on potential racial or ethnic-associated differences [16]. This additional step clarifies the true significance of the findings. Further, while race and ethnicity may be significantly associated with the outcome variable of interest, presentation of results alone may not capture the social nuance of the experience [13]. Research findings can have unintended consequences; therefore, it is important to be mindful of how results using race and ethnicity data are interpreted. Any outcomes based on these concepts can influence how individuals in the group(s) under study are perceived, which may perpetuate stereotypes, discrimination, and prejudice. Research results also impact the resources allocated to certain issues commonly associated with race and ethnicity (e.g. diversity efforts for admissions, recruitment and faculty retention, etc.) [2].

##### Recommendation:

Provide an interpretation of research findings that extends beyond the simple reporting of statistical significance [17]. Use the social context of race and ethnicity variables to assign sufficient attribution to social explanatory factors (e.g., economic, trust, biased context) and, based on the literature, explore potential reasons behind relationships that take the context into account. Explain the effect racial and ethnicity differences have on the dependent variable to avoid assumptions about a particular group or reaching unfounded associations between race or ethnicity and the outcome variable [17]. Refrain from extrapolating findings beyond the study’s sample or conceptualization of race and ethnicity, and make a conscious effort to remove personal biases and/or assumptions from the interpretation.

## Conclusion

Our intent is not to recommend the discontinuation of the use of race and ethnicity variables from medical education research, but rather to challenge researchers to use these variables more intentionally and in their proper context. Education researchers should acknowledge the limitations surrounding race and ethnicity as described with these recommendations. As racial and ethnic categories remain fluid amid changing demographic patterns and sociopolitical perspectives, it is critical that research acknowledges the underlying implications that accompany these variables. How race and ethnicity are conceptualized, operationalized, categorized, and interpreted in research will impact both the intentional and unintentional conclusions drawn from the results. We urge a more thoughtful and systematic use of these concepts to provide results that will inform, rather than adversely impact, ongoing issues in medical education as well as larger society.
